# Bi-directional context-aware network for the nested named entity recognition

**DOI:** 10.1038/s41598-024-67114-6

**Published:** 2024-07-12

**Authors:** Yao Li, Ningsheng Liao, He Yan, Ye Zhang, Xu Wang

**Affiliations:** School of Artificial Intelligence, Chongqing University of Technology, Chongqing, 401135 China

**Keywords:** Nested named entity recognition, Bi-directional context-aware network, Bi-affine mechanism, Rotary position embedding, Computer science, Mathematics and computing

## Abstract

The Span-based model can effectively capture the complex entity structure in the text, thus becoming the mainstream model for nested named entity recognition (Nested NER) tasks. However, traditional Span-based models decode each entity span independently. They do not consider the semantic connections between spans or the entities’ positional information, which limits their performance. To address these issues, we propose a Bi-Directional Context-Aware Network (Bi-DCAN) for the Nested NER. Specifically, we first design a new span-level semantic relation model. Then, the Bi-DCAN is implemented to capture this semantic relationship. Furthermore, we incorporate Rotary Position Embedding into the bi-affine mechanism to capture the relative positional information between the head and tail tokens, enabling the model to more accurately determine the position of each entity. Experimental results show that compared to the latest model Diffusion-NER, our model reduces 20M parameters and increases the F1 scores by 0.24 and 0.09 on the ACE2005 and GENIA datasets respectively, which proves that our model has an excellent ability to recognise nested entities.

## Introduction

**Figure 1:**
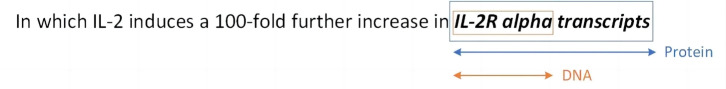
Example diagram of nest named entity.

Named Entity Recognition (NER) is a sub-task of information extraction that aims to locate and classify named entities within text sequences. These entities are typically categorized into predefined groups, such as people’s names, organizations, and locations. NER is fundamental for applications like Relation Extraction^1^, Building Knowledge Graphs^2^, and Question Answering System^3^. The prevalent approach treats NER as a sequence annotation task^4,5^, where each word associated with an entity is labelled with a specific category. However, this approach has difficulties with nested entities, where a word may fall under multiple categories. As shown in Fig. 1, “IL-2R alpha transcripts” is tagged as “protein”, while “IL-2R alpha” is classified as “DNA”. This adds complexity to the NER process.

To tackle the challenge of nested entities, various studies have introduced span-based models^6^. Unlike traditional sequence labeling models, span-based models are better suited for handling cases where entities are nested within each other. Span-based models first convert input sentences into abstract representations, subsequently identifying and characterizing potential entity spans by capturing crucial features. These features help a classifier recognize and categorize nested named entities. However, these models often struggle with accurately defining entity boundaries. To improve this, some researches has included the bi-affine mechanism to enhancing boundary clarity in the span representations.

Despite these advancements, many models still overlook the semantic relationships between spans. Yan et al.^7^ have addressed the issue by employing convolutional neural network (CNN) to analyze score matrices. However, CNN primarily focus on local feature interactions and frequently miss the broader semantic relationships among distant spans. Additionally, these methods commonly ignore the positioning of head and tail tokens within a span. This oversight can result in inaccuracies in determining a span’s length and importance, ultimately leading to incorrect entity recognition.

**Figure 2:**
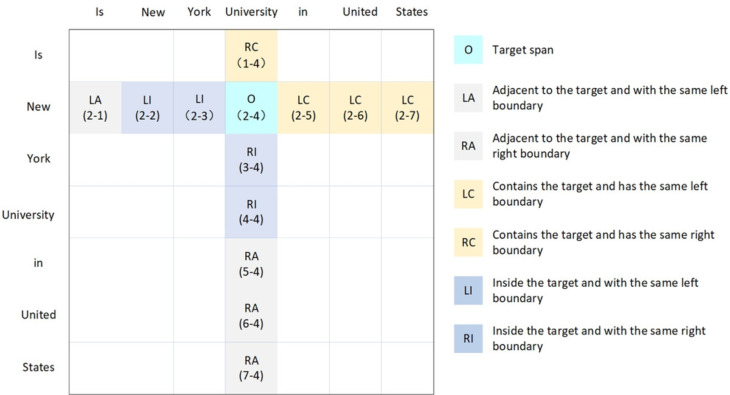
All valid spans in the sentence.

Therefore, we propose a novel model that focuses on inter-span semantic relations. As illustrated in Fig. 2, the model recognizes specific spatial relationships between the position of each span and its neighbors in a planarized sentence representation. Unlike traditional models, the approach facilitates simultaneous consideration of both localized and long-distance semantic relations. Given that the new semantic relationship model displays a cruciform pattern on the plane matrix, we have developed a Bi-Directional Context-Aware Network (Bi-DCAN) to effectively learn and interpret these patterns. The Bi-DCAN is equipped with a multi-granularity cross-attention mechanism. The mechanism effectively tackles the complex dependencies of semantic relationships between spans by capturing both horizontal and longitudinal relationships. Additionally, we have integrated Rotary Position Embedding (RoPE) into the biaffine component. This integration enables the model to effectively capture the relative positional information between the head and tail tokens of entities. Our comprehensive experiments on three publicly accessible nested datasets clearly demonstrate the effectiveness of our proposed model.

The key contributions of this research can be summarized as follows:

- We design a novel relational representation between neighbouring spans. The relation representation has advantages over traditional span-based models in learning information about semantic relationships between spans.
- We propose a Bi-DCAN model based on a self-created multi-granularity cross-focusing mechanism. This mechanism can simultaneously learn the global and local inter-span semantic features, and get rich feature information.
- We propose an improved bi-affine mechanism. This mechanism is used in conjunction with RoPE to efficiently capture the relative location information of entity head and tail tokens.

## Related work

In recent years, many research methods have emerged for the task of nested named entity recognition (Nested NER). These methods can be primarily classified into three categories: Layer-based models, Hypergraph-based models, and Span-based models.

Layer-based models employ hierarchical approaches to identify nested entities across multiple levels. In early research, Ju et al.^8^ utilized a multi-layer neural network for this purpose. The network features neuron layers stacked from the innermost to the outermost, with each layer functioning as a sequential tagging model. On this basis, Fei et al.^9^ introduced a scheduling attention mechanism based on multitask learning that uses different tasks to identify entities with different nested layers. Furthermore, Fisher et al.^10^ developed a novel neural network model that groups annotated entities into a nested structure, assigning a unique label to each entity. In addition to this, some researchers prefer single decoder designs. For example, Wang et al.^11^ devised a neural hierarchical model named “Pyramid,” which processes Nested NER tasks from the innermost to the outermost layers. Shibuya et al.^12^ designed a CRF-based decoding strategy that starts from the outermost entities and progresses inward. This method employs separate CRF layers for each entity category, simplifying the decoding process and eliminating the need for re-encoding.

Hypergraph-based models use a hypergraph structure to describe nested structures in sentences, labelling different named entity tokens via hyperarcs. Initial studies employed directed hypergraphs^13^ to enhance boundary detection and category prediction. These studies emphasized that hyperarcs delineate the relationships among nested entities by connecting multiple nodes, thereby forming paths that lead to distinct subhypergraphs. To tackle the issue of structural ambiguity, Wang et al.^14^ developed a neural segmented hypergraph model. The model combines neural network to generate distributed feature representations and integrates hypergraphs to address structural ambiguities. In a different approach, Katiyar et al.^15^ adopted the BILOU labeling method and introduced a hypergraph structure based on a standard LSTM. The method fully leverages the capabilities of the LSTM technique, enhancing the representation and processing of complex entity relationships within hypergraphs.

Span-based models achieve nested entity recognition by enumerating and classifying spans within a sentence. Initial studies^16^ conducted a comprehensive categorization of all possible spans, utilizing information on both the interiors and boundaries of entities to generate span representations. To minimize the computational complexity associated with span enumeration, subsequent research primarily focused on utilizing entity boundary information for span generation. Zheng et al.^17^ added a boundary detection task to improve span representations. This adjustment overcomes the shortcomings of traditional methods that focus on learning fragmentary representations without explicit boundary supervision. Another study^18^ introduced a boundary detection method that employs token-level classifiers to determine the start and end positions of an entity. These positions are then combined and fed into another classifier for final entity classification.In a different approach, Yu et al.^19^ applied the bi-affine mechanism to classify spans based on their boundary information. Meanwhile, Liu et al.^20^ proposed dividing the NER task into two subtasks: entity recognition and entity classification. To address the oversight of semantic relationships between spans, Yan et al.^7^ introduced a planarized sentence representation. This method merges the head and tail entity representations into a 3D feature matrix and uses a convolutional neural network to enable semantic interactions between spans.

Although the aforementioned method facilitates span interaction to some extent, it predominantly focuses on interactions within locally adjacent regions. We suggest that in a planarized layout, a span has a deeper semantic link with its horizontal and vertical neighbors than with adjacent spans. As illustrated in Fig. 2, spans that are horizontally or vertically aligned typically exhibit shared boundaries or containment relationships within the sentence structure. To explore these connections, we developed a Bi-DCAN based on a multi-granularity cross-attention mechanism to thoroughly understand these semantic relationships. In addition, we add RoPE to the MLP layer of the bi-affine mechanism. This enhancement enables the model to accurately learn the relative position information between head and tail markers through RoPE, thus significantly improving its recognition capabilities.

## Methodology

**Figure 3:**
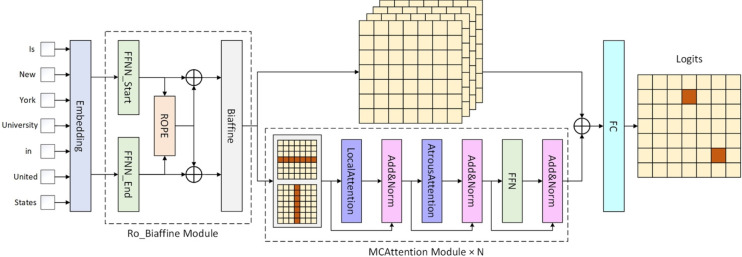
Model structure.

Our model’s workflow is shown in Fig. 3, which consists of two main parts: the boundary information extraction module and the entity semantic perception module. The boundary module adopts an improved bi-affine mechanism to obtain entity boundary information by combining with RoPE. The semantic module uses a Bi-DCAN to gather span representations with contextual semantic details. These representations are combined with the feature matrix from the boundary module. Finally, we use a classifier to classify the entities.

### Encoder layer

At the start of our model, we use the BERT pre-trained model as a sentence encoder. This encoder converts each token in the sentence into an embedding vector, as shown in Eq. (1).

1 $$\begin{aligned} D = \text {BERT}(w_1, w_2, \ldots , w_n) = (d_1, d_2, \ldots , d_n) \end{aligned}$$

where, *D* represents the embedding vector acquired for each sentence, The *n* denotes the number of tokens in the input sentence, $d_i \in \mathbb {R}^l$ denotes the embedding vector of each token, and *l* denotes the dimensionality of the embedded feature.

### Bi-affine attention mechanism augmented with location information

Current span-based models often use the bi-affine mechanism to create a 3D feature matrix that represents semantic relationships within a sentence. However, these models usually ignore the positional information that indicates the exact length of the entity’s span. This includes the relative positions of the sentence’s head and tail tokens. We believe ignoring this detail can lead to a mismatch between the predicted and actual positions of entity heads and tails. The problem ultimately leads to a compromised entity identification capability of the model, especially for long entities.

Therefore, we enable the bi-affine mechanism to capture the relative positions of head and tail tokens by integrating the RoPE module. Specifically, we utilise the MLP layer to process sentence embedding vectors. This produces a head-tail vector representation as shown in Eqs. (2, 3).

2 $$\begin{aligned} D_s= & {} \text {LeakyReLU} (W_sD+b_s) \end{aligned}$$

3 $$\begin{aligned} D_t= & {} \text {LeakyReLU} (W_tD+b_t) \end{aligned}$$

where $W_s\in R^{n\times l}$ and $W_t\in R^{n\times l}$ are two trainable weight matrices, $b_s$ and $b_t$ are the corresponding bias vectors. $D_s\in R^{n\times l}$ and $D_s\in R^{n\times l}$ are the sequence representations of the head and tail spans, *G* denotes the word vector dimension of the head-tail tokens.

However, the basic MLP layer used to derive the head and tail vectors lacks the capability to incorporate relative position information. This limitation arises from the independent computation of head and tail vectors within the MLP layer during projection. Consequently, we address this issue by introducing a position encoding module, which enables us to compute the relative position of the head and tail vectors. This computation yields the relative position information $R_s$ and $R_t$, with dimensions identical to those of $D_s$ and $D_t$ respectively. Drawing inspiration from the approach outlined in^21^, we integrate the relative position information into the bi-affine mechanism as follows:

According to the geometrical significance of complex multiplication, the relative position transformation corresponds to the rotation of the vectors, in the case of a two-dimensional matrix, as shown in Eq. (4).

4 $$f(q_{m} ,m) = \left( {\begin{array}{*{20}c} {{\text{ }}\cos m\theta } & { - \sin m\theta } \\ {\sin m\theta } & {\cos m\theta } \\ \end{array} } \right)\left( {\begin{array}{*{20}c} {q_{m}^{{(1)}} } \\ {q_{m}^{{(2)}} } \\ \end{array} } \right)$$

where $q_m^{(1)},\hspace{5.0pt}q_m^{(2)}$ is the representation of $q_m$ in 2-dimensional coordinates, and $f(q_m, m)$ can represent the positional information embedding of $q_m$. Therefore, based on this principle, it is generalized to a general form, using the header tag vector as an example.

5 $$R_{s} = RoPE(d_{i} ) = \left( {\begin{array}{*{20}c} {d_{0} } \\ {d_{1} } \\ {d_{2} } \\ {...} \\ {d_{{l - 2}} } \\ {d_{{l - 1}} } \\ \end{array} } \right)^\circ \left( {\begin{array}{*{20}c} {\cos m\theta _{0} } \\ {\cos m\theta _{0} } \\ {\cos m\theta _{1} } \\ {...} \\ {\cos m\theta _{{l/2 - 1}} } \\ {\cos m\theta _{{l/2 - 1}} } \\ \end{array} } \right) + \left( {\begin{array}{*{20}c} { - d_{1} } \\ {d_{0} } \\ { - d_{3} } \\ {...} \\ { - d_{{l - 1}} } \\ {d_{{l - 2}} } \\ \end{array} } \right)^\circ \left( {\begin{array}{*{20}c} {\sin m\theta _{0} } \\ {\sin m\theta _{0} } \\ {\sin m\theta _{1} } \\ {...} \\ {\sin m\theta _{{l/2 - 1}} } \\ {\sin m\theta _{{l/2 - 1}} } \\ \end{array} } \right)$$

where, $m\in \left[ 0, m\right)$ represents the position of the token in the sentence, while $\theta _i=10000^{-2i/l},\hspace{5.0pt}i\in \left[ 0,l/2\right)$ signifies the token embedding dimension of the input sequence. Rotary Position Embedding achieves relative positional encoding by incorporating absolute positional encoding, thus merging the benefits of both absolute and relative positional encoding methods.

Finally, we add the relative position information of the head and tail to their respective vectors and input them into the biaffine function for processing. This step yields a position information-enhanced 3D sentence representation matrix, $R_0\in R^{l\times n\times n}$, as shown in Eq. (6), where ’Biaffine’ refers to the biaffine function.

6 $$\begin{aligned} R_0 = \text {Biaffine}(D_s+R_s, D_t+R_t) \end{aligned}$$

### Bi-directional context-aware network

In order to obtain the semantic relationship between a target span and its horizontal or vertical spans, we design a Bi-DCAN. Specifically, we first decompose the obtained 3D matrix is decomposed and divided into $H=\left[ H^1, H^2,..., H^n\right]$ and $V=\left[ v^1, v^2,..., v^n\right]$ according to the horizontal and vertical directions respectively. We set the decomposition width as 1, and since the matrix is a square matrix, the resulting $H^i$ and $V^i$ are standardized as $H^i,\hspace{5.0pt}V^i\in R^{n\times l}$ and $i\in \left[ 0, n\right)$.

**Figure 4:**
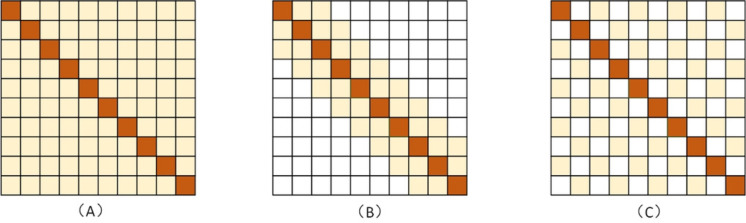
Schematic representation of different attention mechanisms: (**A**) is the ordinary self-attention mechanism, (**B**) is the localized attention mechanism, and (**C**) is the null attention mechanism.

After obtaining the horizontal and vertical feature matrices, we put them into the local and extended attention mechanisms of the multi-granularity attention network for learning. In this way, we were able to efficiently obtain the local semantic dependencies and long-distance dependencies between the spans. A comparison between the local attention mechanism and the dilation attention mechanism with the standard attention mechanism is shown in Fig. 4.

The local attention mechanism targets localized regions near the span in the sequence. To start, we expand the input feature matrix $X\in R^{n\times l}$ to $X^q\in R^{n\times 1\times l}$. We then adjust this according to the local length to get $X^a\in R^{n\times p_s\times l}$, where $p_s=2n+1$. Finally, we compute the local attention using Eqs. (7, 8, 9).

7 $$\begin{aligned} Q_{lo}= & {} X^qW_{lo}^Q \end{aligned}$$

8 $$\begin{aligned} K_{lo}, V_{lo}= & {} X^aW_{lo}^K, X^aW_{lo}^V \end{aligned}$$

9 $$\begin{aligned} \text {Localattention}(X)= & {} \text {softmax}\left( \frac{Q_{lo}K_{lo}^T}{\sqrt{l}}\right) V_{lo} \end{aligned}$$

where $W_{lo}^Q\in R^{n\times 1\times l}$, $W_{lo}^K\in R^{n\times p_s\times l}$ and $W_{lo}^V\in R^{n\times p_s\times l}$ are three learnable weight matrices.

The dilation attention mechanism calculates the attention relationships between a span and its neighboring spans within a specific distance, known as the Dilation Rate. This approach helps us understand the semantic relationships between spans that are far apart. Specifically, we reshape the input matrix. Then, according to the Dilation Rate *dl*, we select one word every *dl* positions. This creates $X^d\in R^{(n/dl)\times l}$. Finally, we perform the dilation attention calculation as outlined in Eqs. (10, 11).

10 $$\begin{aligned} Q_{a}, K_{a}, V_{a}= & {} X^dW_{a}^Q, X^dW_{a}^K, X^dW_{a}^V \end{aligned}$$

11 $$\begin{aligned} \text {Atrousattention}(X)= & {} \text {softmax}\left( \frac{Q_{a}K_{a}^T}{\sqrt{l}}\right) V_{lo} \end{aligned}$$

where $\left\{ W_{a}^Q, W_{a}^K, W_{a}^V\right\} \in R^{(n/dl)\times l}$ are three learnable weight matrices.

We take the horizontal vector as an example. According to the above formula, we can get a horizontal vector representation $H_a^i$ after the multi-granularity cross-attention mechanism. The specific calculation process is shown as follows:

12 $$\begin{aligned} H= & {} \left[ H^1, H^2,..., H^n\right] \end{aligned}$$

13 $$\begin{aligned} H_a^i= & {} \text {Localattention}(H^i) + H^i \end{aligned}$$

14 $$\begin{aligned} H_a^i= & {} \text {Atrousattention}(H_a^i) + H_a^i \end{aligned}$$

15 $$\begin{aligned} H_a= & {} \text {attention}_h(H) = [H_a^1, H_a^2,..., H_a^n] \end{aligned}$$

where $\text {attention}_h()$ denotes the horizontal attention mechanism module, and similarly, the vertical attention computation can be derived as $V_a = \text {attention}_v(V) = \left[ V_a^i, V_a^2, ..., V_a^n\right]$.

We argue that local attention mechanisms help overcome certain limitations of dilation attention mechanisms. Conversely, dilation attention addresses issues in local attention mechanisms that might overlook long-range correlations. Consequently, the multi-granularity cross-attention mechanism provides a balanced solution. It conserves computational memory while effectively capturing both strong local correlations and sparse long-range correlations.

Finally we sum the obtained horizontal and vertical outputs and improve the generalization of the model by one MLP layer. The final 3D matrix with enhanced span context-aware information is obtained $R_a\in R^{n\times n\times l}$.

16 $$\begin{aligned} R_a= & {} \text {Norm}(H_a + V_a) \end{aligned}$$

17 $$\begin{aligned} R_a= & {} R_a + \text {Norm}(\text {MLP}(R_a)) \end{aligned}$$

### Training strategies

Our decoding process is adopted as shown in the paper^7^. Specifically, we use the perceptron to obtain the predicted logits as shown in Eq. (18).

18 $$\begin{aligned} P = \text {Sigmoid}(W_oR_{out}+b) \end{aligned}$$

where $W_o\in R^{T\times l}$, $b\in R^T$, $P\in R^{n\times n\times T}$. *T* is the number of entity categories. Subsequently we use binary cross entropy to calculate the loss as shown in Eq. (19).

19 $$\begin{aligned} L_{BCE} = -\sum _{0\le i,j\le n}y_{ij}\text {log}(P_{ij}) \end{aligned}$$

Since the upper and lower triangles of the scoring matrix are symmetrically related, we calculate the scores of the upper triangular portion of the scoring matrix as in our reasoning:

20 $$\begin{aligned} P^e_{ij} = (P_{ij}+P_{ji})/2 \end{aligned}$$

where $i\le j$. To decode the scores, we initially eliminate non-entity spans, as none of them possess scores exceeding 0.5. Subsequently, we arrange the remaining spans based on their highest entity scores. We choose spans in accordance with this order and disregard spans in situations where their boundaries are in conflict.

## Experimental results and analysis

### Dataset

Our model was evaluated on three publicly available Nest NER datasets: ACE2004^22^, ACE2005^23^, and GENIA^24^. Table 1 lists the statistics of the datasets. Here, ’Total’ refers to the total number of entities, ’Nested’ indicates the number of nested entities, and ’Ratio’ shows the percentage of nested entities in the dataset.

**Table 1:** Dataset specific information.

Category	Train			Eval			Test		
	Total	Nested	Ratio (%)	Total	Nested	Ratio (%)	Total	Nested	Ratio (%)
ACE2004	22204	10149	46	2514	1092	43	3035	1417	47
ACE2005	24411	9389	40	3200	1112	37	2933	1118	39
GENIA	47006	8382	18	4461	818	18	5596	1212	29

We processed the ACE2004 and ACE2005 datasets using the same method described in paper^19^, dividing the data into training, validation, and testing sets.

Likewise, we applied the method from paper^14^ to preprocess the GENIA dataset. It was split into training, validation, and test sets in an 8:1:1 ratio for our experiments.

### Assessment of indicators

We use precision, recall, and F1 scores to evaluate our model and compare it to related works.

### Experimental setup

In the experiments, we utilized a single RTX 4090 GPU for training the model on Windows 10. The experimental setups for the GENIA dataset and ACE dataset are presented in Tables 2 and 3.

**Table 2:** Experimental parameters for GENIA.

Hyper-parameters	Value
Pre-trained models	BioBERT-base^25^
Dropout	0.5
Learning rate	7e−6
Batchsizes	8
biaffine_size	400
Epoch	10
heads of attention mechanisms	8

**Table 3:** Experimental parameters for ACE.

Hyper-parameters	Value
Pre-trained models	BERT-large
Dropout	0.5
Learning rate	2e−5
Batchsizes	48
biaffine_size	200
Epoch	50
heads of attention mechanisms	4

### Hyperparametric analysis

**Figure 5:**
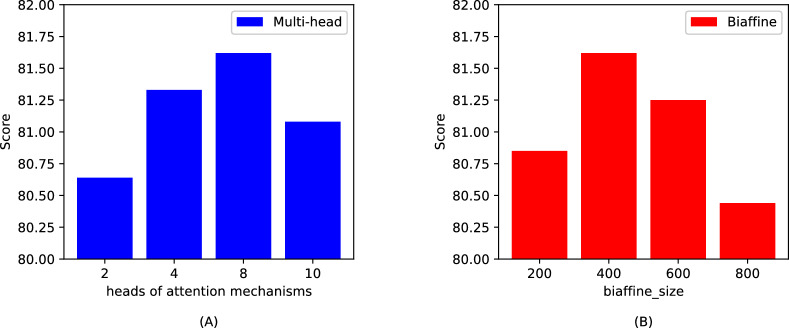
Experimental results on the GENIA dataset: (**A**) is the number of; (**B**) is biaffine_size.

**Figure 6:**
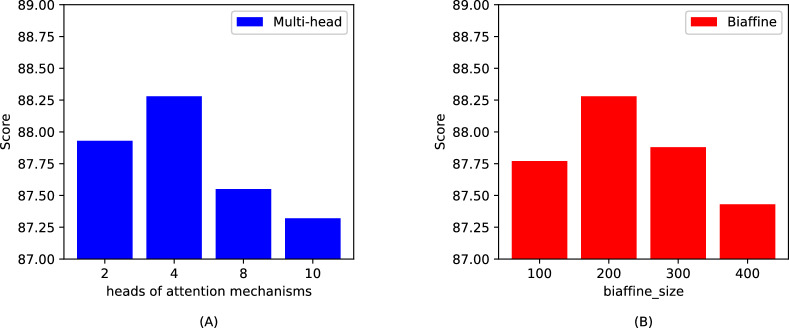
Experimental results on the ACE2004 dataset: (**A**) is the number of attention mechanism heads; (**B**) is biaffine_size.

In this section, we aim to evaluate the appropriateness of the hyperparameter settings by conducting a series of experiments on two datasets, GENIA and ACE2004. Specifically, we focus on analyzing the impact of two key hyperparameters: the biaffine size and the number of attention heads in our model. This analysis involves treating these hyperparameters as control variables to systematically explore their effects on the model’s performance. Detailed results from these experiments are depicted in Figs. 5 and  6, which illustrate the outcomes and trends derived from varying these parameters.

Through our analysis, we observed that the GENIA dataset is characterized by an abundance of technical terms and complex vocabulary. This complexity demands that the model possess an enhanced feature learning capability. Consequently, a larger number of attention mechanism heads and an increased biaffine_size are necessary to adequately capture the intricate patterns within this dataset. In contrast, the ACE2004 dataset presents simpler content and a smaller volume of data, which could potentially lead to overfitting if larger parameter values are used. Therefore, it is prudent to opt for smaller hyperparameter settings for this dataset to avoid overfitting and ensure that the model generalizes well across different types of textual data.

### Comparison experiment

In this section, we perform experiments with the ACE2004, ACE2005, and GENIA datasets to compare our model with span-based models from related works. The results are presented in Table 4. The best results are highlighted in bold.

**Table 4:** Experimental parameters for ACE.

Model	ACE2004			ACE2005			GENIA		
	Pr.	Rec.	F1	Pr.	Rec.	F1	Pr.	Rec.	F1
Biaffine^19^	87.3	86.0	86.7	85.2	85.6	85.4	81.8	79.3	80.5
MRC^26^	85.05	86.32	85.98	**87.16**	86.59	86.88	81.14	76.82	78.82
Loc &Label^27^	87.44	87.38	87.41	86.09	87.27	86.67	80.19	80.89	80.54
SpanGCN^28^	86.70	85.93	86.31	84.37	85.87	85.11	77.92	80.74	79.30
lc-trees^29^	87.39	88.40	87.90	85.97	87.87	86.91	−	−	−
BS^30^	**88.43**	87.53	87.98	86.25	88.07	87.15	−	−	−
Triaffine^31^	87.13	87.68	87.40	86.70	86.94	86.82	80.42	**82.06**	81.23
W2NER^32^	87.33	87.71	87.52	85.03	**88.62**	86.79	83.10	79.76	81.39
BR^33^	−	−	−	86.88	84.83	85.84	80.81	77.43	79.09
CNN-NER^7^	87.82	87.40	87.61	86.39	87.24	86.82	**83.37**	79.43	81.35
Diffusion-NER^34^	88.11	88.66	**88.39**	86.15	87.72	86.93	82.10	80.97	81.53
GAT-NER^35^	87.17	87.40	87.28	85.71	86.23	85.97	79.48	80.01	79.74
STM-NER^36^	−	−	−	87.11	87.14	87.12	79.02	80.68	79.87
Ours	88.02	**88.68**	88.28	86.76	87.96	**87.36**	82.68	80.60	**81.62**

Best values are in bold.

Notably, on the ACE2005 and GENIA datasets, our model demonstrates superior performance in terms of F1 scores, surpassing the 87% threshold on the ACE2005 dataset and exceeding 81% on the GENIA dataset. Moreover, concerning the ACE2004 dataset, our model exhibits marginal deviation from the optimal Diffusion-NER by a mere 0.11. These findings underscore the efficacy of our proposed model.

### Ablation experiment

In this section, we perform ablation experiments to analyze the effect of different modules on the model performance by removing the module. As shown in the Table 5, all the modules contribute to the improvement of the model performance.

**Table 5:** Dataset specific information.

Model	ACE2004	ACE2005	GENIA
Defualt	88.28	87.36	81.62
w/o RoPE	87.95	87.17	80.80
w/o Bi-DCAN	87.67	86.91	81.12
w/o RoPE & Bia	86.98	86.48	80.28
w/o RoPE & Bi-DCAN	87.13	86.61	80.18
w/o RoPE & Bi-DCAN & Bia	86.66	86.17	79.85

Firstly, the RoPE enables the head and tail tokens obtained by the Biaffine mechanism to carry relative position information, which effectively alleviates the entity boundary recognition ambiguity problem. Second, the Bi-DCAN enriches the feature information by learning the semantic dependencies between spans in the planarised sentence features, which improves the model performance. This suggests semantic relationships between spans that share horizontal and vertical coordinates with the target span. Learning this semantic information enriches the semantic representation of spans.

### Semantic dependency strength analysis between span

**Figure 7:**
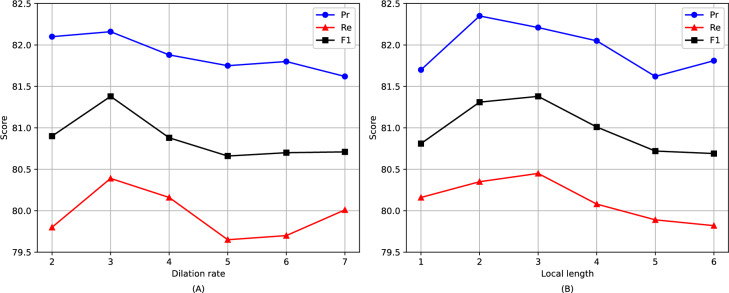
Experimental results on the GENIA dataset: (**A**) are for different dilation rates; (**B**) are for different local lengths.

In this section, we use the GENIA dataset to investigate the strength of semantic dependencies between different distances spans. We conduct studies with different dilation rates and local lengths. To highlight the results, we remove the local attention layer in experiments with varying dilation rates, and likewise, we remove the dilation attention layer in experiments with varying local lengths. The results are presented in Fig. 7.

From (A), we notice that the dilation rate reaches its peak at 3 before decreasing. This indicates that the attention mechanism performs best when the relative distance between spans is 3, where the semantic dependency strength is highest.

From (B), we observe that the performance is optimal when the local length is 3, indicating the strongest semantic dependency between a span and its neighboring spans within a distance of 3.

These experiments illustrate that semantic dependencies between target spans are stronger when they are closer to spans located on the same horizontal and vertical coordinates, and weaken as the distance increases.

### Experimental comparison of different entity lengths

In this section, we evaluate the model’s performance in recognizing entities of different lengths, with a focus on its ability to identify entity boundaries and types for long entities. We performed experiments using the GENIA dataset and selected entities of length 4, 5, and greater than 5 from the test set to calculate F1 values. To demonstrate the effectiveness of the RoRE module in identifying long entities, we compare our model with CNN-NER, Diffusion-NER, W2NER, and w/o RoPE. The CNN-NER utilizes convolutional modules to capture local positional information. The Diffusion-NER employs diffusion models to identify entity boundaries by generating and optimizing noise. The W2NER uses traditional positional encoding to obtain positional information. Experimental results are presented in Table 6.

**Table 6:** Comparative experimental results for different entity lengths.

Model	E = 3	E = 4	E > 4
Our	85.57	80.61	77.59
w/o Rope	83.53	78.64	74.44
CNN-NER	84.97	80.02	76.11
Diffusion-NER	85.36	80.14	77.02
W2NER	85.18	79.84	76.14

As shown in Table 6, when the entity length is 3, our model exhibits comparable performance to other baseline models. And as the entity length increases, the advantage of our model gradually comes out. Experimental results demonstrate that introducing RoPE into Biaffine to enhance positional information guidance significantly improves the accurate representation of the head and tail tokens of long entities by the model.

### Experimental results for different nested models

In order to comprehensively analyze the performance of our model in identifying nested entities, we conducted experiments following the experimental setup outlined in reference^37^. In this setup, nested entities are structured into tuples, where each tuple comprises an outer entity (i.e., the longest entity) along with several inner entities (entities nested within the outer entity). We selected the five most representative entity types for experimentation on the GENIA dataset and calculate their F1 values. The experimental results are presented in the Table 7. Here, “Flat” denotes statistics for non-nested entity recognition, “Nested” indicates statistics for the recognition of each nested entity, “Inner” and “Outer” respectively represent statistics for the recognition of inner and outer entities within the tuple, and finally, “Nesting” denotes statistics for the simultaneous recognition of the entire tuple.

**Table 7:** Experimental results for different nesting types.

Model	Flat	Nested	Inner	Outer	Nesting
Our	84.04	**67.02**	**52.68**	81.18	**40.77**
W2NER	84.27	65.66	50.21	80.43	38.46
CNN-NER	83.87	66.23	50.69	80.65	38.74
Diffusion-NER	**84.55**	66.84	51.62	**81.34**	39.44

Best values are in bold.

The Table 7 indicates that our model performs well in all the complex nested environment experiments. The analysis suggests that “flat” entities are less difficult to recognise and have larger data volumes, thus narrowing the performance differences between models. The “outer” entities have smaller data volumes, and the diffusion model has some advantages for small sample experiments. Nevertheless, our model still shows strong overall performance. These experimental results effectively validate the competitive advantage of our model in the Nest NER task.

### Analysis of the model training process

In this section, we study the training process of the model using the GENIA dataset as an example and training the model using BioBERT-base. We compare the computational efficiency of our model with related works, as illustrated in Table 8.

**Table 8:** Number of parameters for different models.

Model	Params	T(s)	V(it/s)
Our	113M	106	23.96
W2NER	112M	107	23.75
CNN-NER	111M	99	25.61
Diffusion-NER	133M	254	10.01
Triaffine	526M	−	−

As can be seen from the Table 8, the Diffusion-NER employs a diffusion model for entity boundary recognition, which results in a higher number of parameters due to the complexity of the denoising step. The Triaffine model utilises a triple affine attention mechanism for entity recognition, which involves more levels of affine transformations to obtain richer features. In contrast, our model utilises semantically dependent information related to span location relationships to enhance span semantic representations. Since only the attention mechanism is used to compute the 3D feature matrix, the number of parameters of our model is small. These results demonstrate that our model is competitive in terms of computational efficiency.

**Figure 8:**
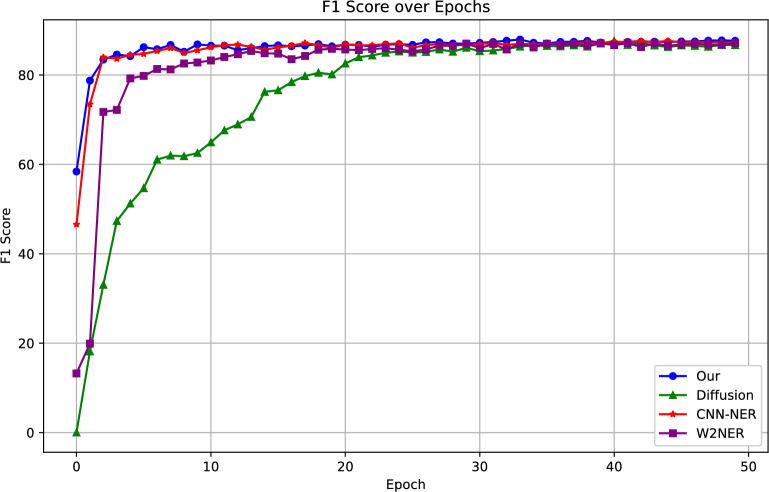
Changes in F1 curves for the validation set during training for the ACE2004 dataset.

Then, we collect the training results of our model on the ACE2004 dataset with the benchmark models CNN-NER and Diffusion-NER. The training performance of the model is analyzed by comparing the curve fit with 50 epochs. The comparison results are shown in Fig. 8.

We run 50 epochs on the ACE2005 dataset and collect the F1 scores for each epoch in the training set, as depicted in Fig. 8. The curves show that our model fits rapidly in the initial training phase, steadily increases around the 3th epoch, and plateaus approximately at the 10th epoch. We retain the best-performing model for test set evaluation.

As illustrated in the Fig. 8, Diffusion-NER exhibits a slower learning fitting speed due to the increased complexity of the network resulting from the introduction and handling of noise during its generation and optimization processes. In contrast, CNN-NER, W2NER, and our model demonstrate lower complexities, thereby facilitating a faster learning fitting rate. Specifically, both CNN-NER and W2NER use simple CNN architectures, which leads them to be relatively poor at capturing long-range dependencies. As a result, they have a slightly slower learning fit compared to our model. consequently leading to a slightly slower learning fitting pace when juxtaposed with our model. The comparison results of the curves confirm the superior convergence speed and stronger learning performance of our model.

### Impact of different pre-training models

To verify the scalability of our method, we select four different pre-training models: BERT-Small, BERT-base, BERT-large, and RoBERTa-base. We conducted experiments on the ACE2004 dataset. As shown in Table 9, our model achieves an F1 value of over 85% across different pre-training models, indicating superior performance. This confirms the scalability of our method.

**Table 9:** Experimental results for different pre-trained models F1 values.

Pre-train model	ACE2004
Bert-small	85.04
Bert-base	86.20
Bert-large	88.28
Roberta-base	87.97

## Conclusion

Aiming at the problem that traditional spanning models cannot effectively obtain the semantic relationship information between spans and the location information of entities, we introduce a Bi-Directional Context-Aware Network for nested named entity recognition. Initially, we propose a novel span-level semantic relation model to represent both local and long-range semantic relations between spans. Subsequently, we present a Bi-DCAN that utilizes a multi-granularity cross-attention mechanism to capture and learn the semantic relation information. Additionally, we utilize RoPE to optimize the bi-affine mechanism, enabling it to effectively leverage positional information. We evaluated our model on three nested datasets. The experimental findings show that our model has fewer parameters, high computational efficiency, and achieved the highest F1 scores on the ACE2005 and GENIA datasets. This demonstrates our model’s excellent capability in nested entity recognition. In future work, we aim to further research the semantic relations between spans in the span model and explore more effective ways to integrate local dependencies and boundary information to enhance the model’s performance even further.

## Data Availability

The datasets analyzed during this study are available at ACE2004 (https://catalog.ldc.upenn.edu/LDC2005T09), ACE2005 (https://catalog.ldc.upenn.edu/LDC2006T06) and GENIA (http://www.geniaproject.org/genia-corpus).
